# Cross‐Range Comparison of Supraglacial Microbial Communities Between Gongga and Gangrigabu Mountains, Southeast Tibetan Plateau

**DOI:** 10.1002/ece3.74278

**Published:** 2026-09-06

**Authors:** Yang Hu, Qiao Liu, Xuyang Lu

**Affiliations:** ^1^ Institute of Mountain Hazards and Environment Chinese Academy of Sciences Chengdu Sichuan China

**Keywords:** climate change, debris‐covered glacier, distance‐decay, high‐throughput sequencing, microbial diversity, Tibetan Plateau

## Abstract

The supraglacial environment represents a unique habitat that harbors overlooked biodiversity, which is increasingly threatened by climate warming but remains underexplored. This study aims to investigate bacterial and fungal distribution patterns on five debris‐covered glaciers from two typical mountain ranges dominated by a monsoon climate (Mt. Gongga and Mt. Gangrigabu). High‐throughput amplicon sequencing of the bacterial 16S rRNA gene (V3–V4 region) and the fungal ITS region was performed using the Illumina MiSeq platform. Despite harsh and nitrogen‐limited conditions, the supraglacial debris across two mountain ranges supported phylogenetically diverse microbial taxa. Dominant bacterial families included *Sphingomonadaceae*, *Chitinophagaceae*, and *Comamonadaceae*, while dominant fungal families included *Thelephoraceae*, *Mortierellaceae*, and *Inocybaceae*, highlighting the establishment of specialized microbial assemblages in oligotrophic glacier surface environments. Compared with Mt. Gongga, supraglacial debris from Mt. Gangrigabu exhibited significantly lower bacterial alpha diversity, likely driven by environmental filtering associated with higher pH and total carbon content. In contrast, fungal alpha diversity showed limited regional variation. Notably, we observed a significant “distance decay” pattern in both bacterial and fungal communities, which illustrated a decline in taxonomic similarity with increasing geographic distance across glaciers, particularly pronounced in bacterial communities (*R* = −0.70, *p* < 0.001). Microbial community composition exhibited stronger glacier‐specific differentiation, heterogeneous environmental selection emerged as the dominant ecological process shaping microbial turnover across isolated glaciers, with pH and carbon content playing dominant roles in shaping microbial community variation. Our cross‐range comparison reveals that deterministic environmental filtering among geographically isolated glaciers governs the spatial turnover in supraglacial microbial communities, providing new insights into the ecological mechanisms governing microbial biogeographic patterns in rapidly changing cryospheric ecosystems across the southeastern Tibetan Plateau.

## Graphical abstract

We investigated bacterial and fungal community distribution on supraglacial debris from two monsoon‐influenced mountain ranges (Gongga and Gangrigabu) using high‐throughput sequencing. Despite harsh, nitrogen‐limited conditions, diverse microbial taxa were detected, with clear distance–decay patterns and strong glacier‐specificity; heterogeneous environmental selection emerged as the dominant ecological process shaping microbial turnover across isolated glaciers, with pH and carbon content playing dominant roles in shaping microbial community variation.

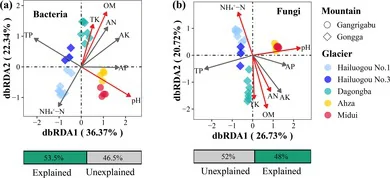

## 1 Introduction

Elucidating the spatial distribution patterns of microbial communities constitutes a fundamental objective in microbial ecology and is critical for understanding the alpine biodiversity and ecosystem processes. In biodiversity studies for a variety of organisms and ecosystems, the similarity of biological communities declines with increasing geographic distance, known as distance‐decay, has been well documented (Basham et al. 2019; Dehling et al. 2020; Morgan et al. 2020; Levy 2024). In recent years, the distance‐decay patterns have also been increasingly reported in microbial communities from different terrestrial ecosystems (Liu et al. 2023, 2024; Zhu et al. 2024). Owing to the small size and large popularity, microorganisms possess a capacity for dispersal widely, potentially weakening the classical distance‐decay relationships described for macroorganisms (Clark et al. 2021). The “everything is everywhere, but the environment selects” hypothesis posits that microorganisms are capable of achieving widespread distribution globally, with local establishment primarily constrained by environmental filtering (O'Malley 2008). Both geographic isolation and environmental heterogeneity interact in complex ways to shape microbial biogeography in terrestrial ecosystems (Morlon et al. 2008; Graco‐Roza et al. 2022).

Glaciers, characterized by persistently low temperatures and nutrient limitation, have long been considered as an unfavorable habitat for life to survive. Advances in microbiological analytical techniques, such as high throughput and metagenomic sequencing, have revealed a surprisingly high diversity of microbial taxa within glacier ecosystems (Liu et al. 2015, 2017), especially considering that these extreme environments exert strong selective pressures, favoring a specific, cold‐tolerant biota (Kim et al. 2022; Ezzat et al. 2025). For instance, bacterial assemblages in the geographically isolated Arctic and Antarctic regions exhibit greater taxonomic similarity (32% overlap) to each other than to those in adjacent temperate regions (Kleinteich et al. 2017). Harsh climatic conditions and pronounced spatial isolation among individual glaciers may drive the formation of highly specialized microbial assemblages, substantially harboring a significant proportion of endemic taxa (Pittino et al. 2023; Ezzat et al. 2025). Anyway, in glacier ecosystems, the microbial communities are relatively under‐explored compared to other ecosystems, largely due to the inaccessibility of many glaciers and challenges in surface sampling (Kim et al. 2022), making it difficult to fully understand the spatial distribution of microbial diversity. Cryoconite holes are among the most extensively studied microbial habitats in glacier ecosystems. These meltwater‐filled depressions on glacier‐ice surfaces (debris‐free glaciers) harbor diverse microbial communities and represent important biological hotspots within the harsh glacial environment (Liu et al. 2017; Pittino et al. 2018, 2023).

Debris‐covered glaciers in China accounted for 11.5% of the total glacier area in 2017 (Zhang and Liu 2017) and increased to 17.2% in 2023 (Yang et al. 2025), due to accelerated ‐ glacier thinning and retreat under climate warming (Brown and Brock 2023). This process supplies greater volumes of debris material from surrounding slopes to the ice surface (Fleischer et al. 2021). On debris‐covered glaciers, previous studies isolated cryophilic taxa such as *Arthrobacter* and *Sphingomonas*, commonly observed in glacier environments (Liu et al. 2015, 2018). Besides, supraglacial debris also serves as a unique substrate that traps organic matter and nutrients from the surrounding environment (Hu et al. 2023; Heather et al. 2024). Consequently, it provides potential niches for diverse and metabolically active microbial communities, including soil‐dwelling taxa, vascular plants, even trees (Caccianiga et al. 2011; Azzoni, et al. 2015; Tampucci et al. 2017; Fickert et al. 2022). The supraglacial debris thus creates a unique habitat harboring highly diverse biodiversity that is increasingly threatened by climate warming. Most existing research, however, has focused on characterizing the bacterial taxonomic composition within individual glaciers, including the terrestrial environment and aquatic habitats, such as sediment and supraglacial ponds (Franzetti et al. 2013; Franzetti et al. 2020; Heather et al. 2020). Furthermore, microbial communities exhibit a clear successional chronosequence on the supraglacial debris layer, taxonomic composition shifts from autotrophic to heterotrophic dominance, while community complexity increases toward the glacier terminus within thick, slow‐moving debris (Franzetti et al. 2013; Darcy et al. 2017; Hu et al. 2023).

The southeastern Tibetan Plateau is distributed with many temperate glaciers, which are highly sensitive to climate change (Fugger et al. 2022). Many of them are debris covered and are experiencing accelerated ice loss in recent years under increasing atmospheric temperatures and summer precipitation (Wang et al. 2021). Nevertheless, knowledge regarding the spatial distribution of microbial communities remains limited. This study aims to explore the diversity pattern (alpha and beta diversity) of bacterial and fungal communities and their determinants across five glaciers: three on Mt. Gongga (Dagonga Glacier, Hailuogou No. 1 Glacier, and Hailuogou No. 3 Glacier) and two on Mt. Gangrigabu (Azha Glacier and Midui Glacier). The above glaciers are characterized by dark ice tongues due to debris covered, forming complex microhabitats on the ice surfaces (Heather et al. 2020; Miles et al. 2020; Fugger et al. 2022; Hu et al. 2023). We address the following questions: (1) Do microbial communities exhibit significant differences in diversity and composition across individual glaciers within Mt. Gongga and Mt. Gangrigabu regions? (2) Which factors predominantly govern the spatial distribution of bacterial and fungal communities in supraglacial environments? By comparing five debris‐covered glaciers across the Gongga and Gangrigabu mountains, this study will provide insights into whether supraglacial microbial biogeographic patterns are regionally consistent or regulated by local environmental conditions.

## 2 Materials and Methods

### 2.1 Study Sites and Sample Collection

Field investigations and sample collections of supraglacial debris were conducted during the summer months (June to August) of the year in 2020 and 2021. In the Mt. Gongga region, the Hailuogou Glacier No. 1 (H1, 29°34′ N, 101°59′ E) extends 13.1 km with an area of 24.5 km^2^, with a terminus elevation at 2991 m a.s.l. Hailuogou Glacier No. 3 (H3), an upstream tributary of H1 that has become disconnected from the main glacier, is a smaller glacier less than 5 km in length. The Dagongba Glacier (D, 29°34′ N, 101°48′ E) is 10.6 km long, covers 19.1 km^2^, and the terminus elevation is around 3965 m a.s.l. In the Mt. Gangrigabu region, the Azha Glacier (A, 29°05′ N, 96°48′ E) is 16.7 km long and 13.7 km^2^ in area. Midui Glacier (M, 29°27′ N, 96°30′ E) covers 29.9 km^2^, consisting of two tributaries (10.6 km and 8.2 km long), with a terminus elevation at around 3820 m a.s.l. Overall, all glaciers are representative of monsoonal temperate glaciers that are undergoing pronounced mass loss under current climatic warming; all those glaciers are covered by a continuous layer of debris in their ablation zone.

**FIGURE 1:**
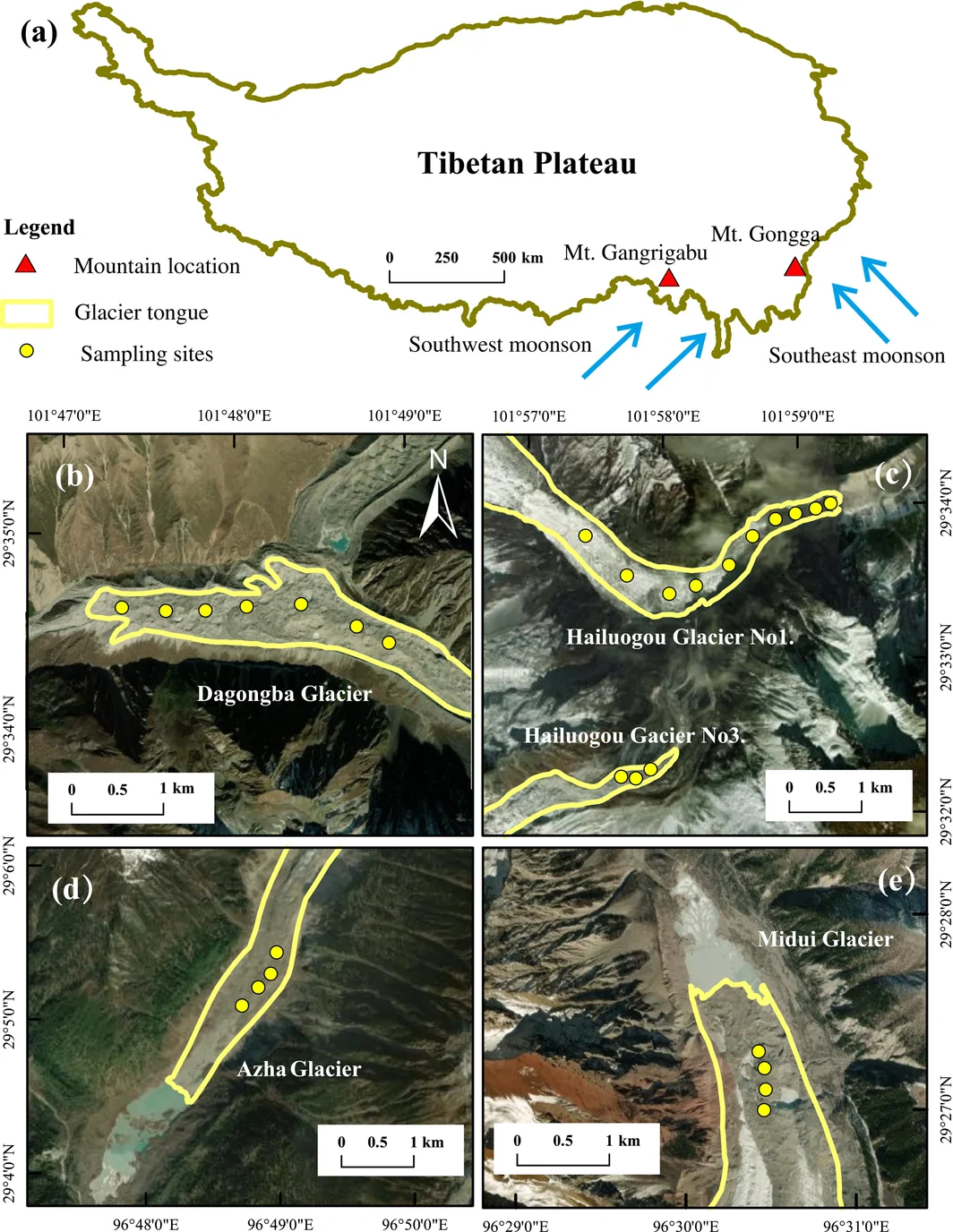
Location of the study area and distribution of sampling sites on the glacier surface. The location of Mount Gongga and Mount Gangrigabu on the southeastern Tibetan Plateau (a). Sampling site distributions on the glacier tongue, including Dagongba Glacier (b), Hailuogou No. 1 Glacier and Hailuogou No. 3 Glacier (c), Azha Glacier (d), and Midui glaciers (e).

Based on site accessibility and glacier size, a total of 28 sites were set up (Figure 1). Specifically, 10 sites were distributed along H1 glacier, 3 sites were selected on H3 glacier, 7 sites were established on D glacier, and 4 sites were set up on each of the A and M glaciers. We collected 28 debris samples from the glacier surface using sterile tools. At each sampling site, five randomly selected subsamples within a 10 m × 10 m plot were collected after removing visible large stones and plant roots, and then homogenized to obtain a composite sample. No sieving was performed to preserve the original microbial community composition. Approximately 20 g of debris sample was transferred into two sterile 10 mL centrifuge tubes for DNA extraction and immediately stored at −20°C. The extracted DNA was subsequently stored at −80°C until further analysis. An additional ~200 g of debris sample was collected in sterile zip‐lock bags for physicochemical analyses. All samples were transported to the laboratory within 15 days.

### 2.2 Physicochemical Analysis

The physicochemical parameters of the debris sample were determined following the standard soil analytical protocols based on Lu (1999) and Bao (2000). The pH value was obtained by preparing a 1:2.5 soil‐to‐water suspension and measuring it using a calibrated digital pH meter (20, Mettler‐Toledo, China). An elemental analyzer (Vario TOC cube; Elementar, Germany) was applied to analyze the total nitrogen (TN), total carbon (TOC), and organic matter (OM) content was estimated from TOC using the conversion factor of 1.724 (OM = TOC × 1.724). The colorimetric determination with a UV–visible spectrophotometer (UV‐1800, Shimadzu, Japan) was used to measure the inorganic nitrogen forms, including nitrate nitrogen (NO_3_^−^‐N) and ammonium nitrogen (NH_4_^+^‐N). Alkali‐hydrolyzable nitrogen (AN) was measured by alkaline hydrolysis following the procedure of Chen et al. (2016). Total phosphorus (TP) and available phosphorus (AP) were assessed by the Mo‐Sb anti‐spectrophotometric method after alkali fusion and sodium hydrogen carbonate solution, respectively. About the total potassium (TK), we measured using atomic absorption spectrophotometry after hydrofluoric acid and perchloric acid digestion. The available potassium (AK) was measured using an inductively coupled plasma atomic emission spectrometer (ICPS‐7500, Shimadzu, Kyoto, Japan). All results were expressed on a dry weight basis.

### 2.3 DNA Extraction and Sequencing Analysis

Total DNA was extracted from 0.5 g debris sample using MagPure Soil DNA LQ Kit (No. D6356‐02, Angen Biotech Co. Ltd). Before PCR amplification, the DNA was verified using agarose gel and NanoDrop, then used as a template after adding barcoded primers and Tks Gflex DNA Polymerase (Takara). The bacterial V3–V4 region of the 16S rRNA gene and the fungal ITS1 region were amplified using universal primers (Supporting Informations, Table S1). A two‐step PCR strategy was employed for both bacterial and fungal amplicon sequencing. The first PCR was used to amplify the target gene region, whereas the second PCR attached sequencing adapters and sample‐specific indices (Supporting Informations, Table S2). AMPure XP beads product by Beckman Coulter (Brea, CA, USA) was then used to purify PCR products. The resulting amplicons were quantified using a Qubit dsDNA Assay Kit (Cat. No. Q32854, Life Technologies, Grand Island, NY, USA). Purified and quantified products were sequenced using the Illumina MiSeq platform (San Diego, CA, USA) at OEbiotech located in Shanghai, China.

Quality control of sequencing data (Fastq format) was performed with Trimmomatic, which removes ambiguous bases (N) according to the publication by Bolger et al. (2014). Then, based on a sliding window method, the low‐quality reads were filtered. For paired‐end reads, both reads were retained only when they passed the quality filtering criteria; otherwise, the read pair was discarded. Subsequently, FLASH was used to merge the paired‐end reads with overlap limits of 10–200 bp, and a maximum mismatch rate of 20% (Magoč and Salzberg 2011). Subsequent denoising was implemented with QIIME (v1.8.0) (Caporaso et al. 2010), where reads containing ambiguous bases, homopolymers, or having a length under 200 bp were excluded, whereas sequences with at least 75% of bases above Q20 were kept. Chimeric sequences were identified and removed using UCHIME (v2.4.2) (Edgar et al. 2011). High‐quality reads were further processed by removing primer sequences, then VSEARCH was employed to cluster the sequences as OTUs (operational taxonomic units) under a 97% sequence similarity (Rognes et al. 2016). Taxonomic assignment was performed using the RDP Classifier against the SILVA SSU r138.1 reference database for bacterial sequences, and against the UNITE database for fungal ITS1 regions, with a confidence threshold of 70%.

### 2.4 Statistical Analysis

This study used R Studio (version 4.1.2) to perform statistical analyses and visualization (R Core Team 2021). To evaluate the differences in supraglacial debris physicochemical properties and microbial alpha diversity among glaciers, we applied the “kruskal” function from the agricolae package (Mendiburu 2023) in R to run the Kruskal–Wallis test, and *p* values were adjusted through the using the Benjamini‐Hochberg (BH) false discovery rate (FDR) method. Using the “vegan” package (version 2.6‐4) (Oksanen et al. 2022), the Non‐metric multidimensional scaling (NMDS) was performed with the metaMDS functions based on Bray‐Curtis distance, and analysis of similarity (ANOSIM) was conducted via the “anosim” functions. Additionally, PERMDISP (permutational analysis of multivariate dispersions) was performed to assess the homogeneity of multivariate dispersion among glacier groups, using the “betadisper” function. Furthermore, the distance‐based redundancy analysis (db‐RDA) was performed using the “capscale” function to assess the relationships between microbial community composition and environmental variables based on Bray‐Curtis dissimilarities. Environmental variables were standardized prior to analysis, and multicollinearity among predictors was evaluated using variance inflation factors (VIF). The significance of the db‐RDA model and individual environmental variables was tested using permutation tests (999 permutations). The correlation between the environmental distance, geographic distance, and microbial community distance (Bray‐Curtis) was achieved through the “stat_cor” function in the Package “ggpubr” (Kassambara 2025). Prior to this step, the environmental distance was calculated using nine variables (pH, TN, TC, OM, NO_3_^−^‐N, NH_4_^+^‐N, TP, TK, AN, AP, and AK) based on Euclidean distance, conducted by the “dist” function in the “stats” package. Geographic distance was calculated between the sampling locations using geographic coordinates, using the “distm” function in the “geosphere” package (Hijmans 2024). The correlations between microbial diversity index and debris physicochemical properties were analyzed and visualized via Mantel tests and correlation heatmaps, respectively, using the vegan and pheatmap packages (Kolde 2025).

## 3 Result

### 3.1 Supraglacial Debris Physicochemical Properties

**FIGURE 2:**
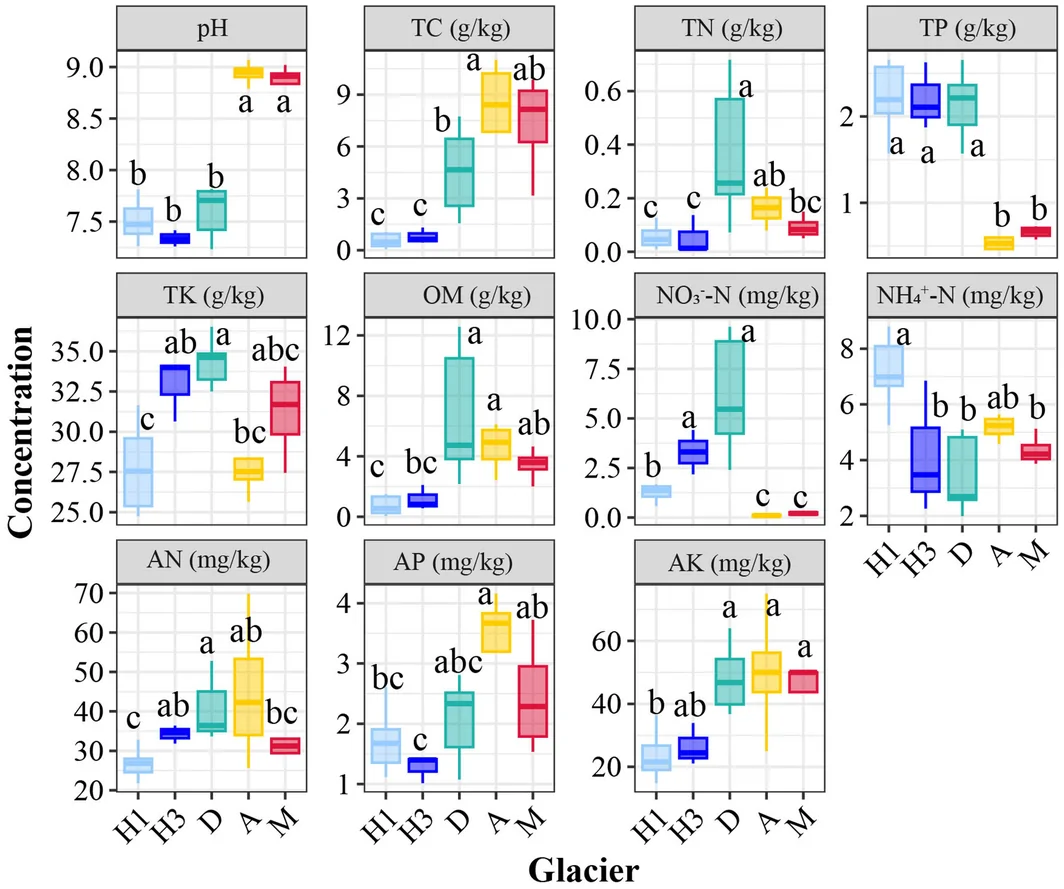
The physicochemical properties of supraglacial debris of different glaciers. Different lowercase letters above the boxes indicate significant differences among glaciers. The Kruskal–Wallis test was performed using the “kruskal” function in the R package agricolae, with *p* values adjusted by the Benjamini–Hochberg (BH) method. Statistical significance was determined at *p* < 0.05. H1, H3, D, A, and M denote Hailuogou No. 1 Glacier, Hailuogou No. 3 Glacier, Dagongba Glacier, Azha Glacier, and Midui Glacier, respectively. H1, H3, and D belong to the Gongga Mountains, whereas A and M belong to the Gangrigabu Mountains.

The physicochemical properties of the 28 supraglacial debris samples (Figure 2) showed that the debris was alkaline, with pH values ranging from 7.23 to 9.07. The TC content ranged from 0.06 to 11.01 g·kg^−1^, TN from 0.01 to 0.71 g·kg^−1^, TP from 0.46 to 2.66 g·kg^−1^, and TK from 18.08 to 36.52 g·kg^−1^. The OM content of supraglacial debris ranged between 0.05 and 12.56 g·kg^−1^. Concentrations of NO_3_^−^–N and NH_4_^+^–N varied from 0.07 to 9.62 mg·kg^−1^ and 1.99 to 9.50 mg·kg^−1^, respectively. The AN, AP, and AK concentrations of supraglacial debris ranged from 21.84 to 69.83 mg·kg^−1^, 1.02 to 4.16 mg·kg^−1^, and 11.19 to 75.00 mg·kg^−1^, respectively. Overall, the supraglacial debris exhibited low N content, resulting in a high C/N ratio. In contrast, due to limited chemical weathering, the debris contained relatively abundant mineral nutrients, particularly P and K. The pH value of supraglacial debris was significantly higher in the glaciers on Mt. Gangrigabu (A and M) than in glaciers on Mt. Gongga (H1, H3, and D), whereas TP and NO_3_^−^–N concentrations showed the opposite pattern. The concentrations of TC, TN, and OM in supraglacial debris were higher in the Dagongga (D) and Azha (A) glaciers than in the other glaciers.

### 3.2 Supraglacial Debris Microbial Composition

**FIGURE 3:**
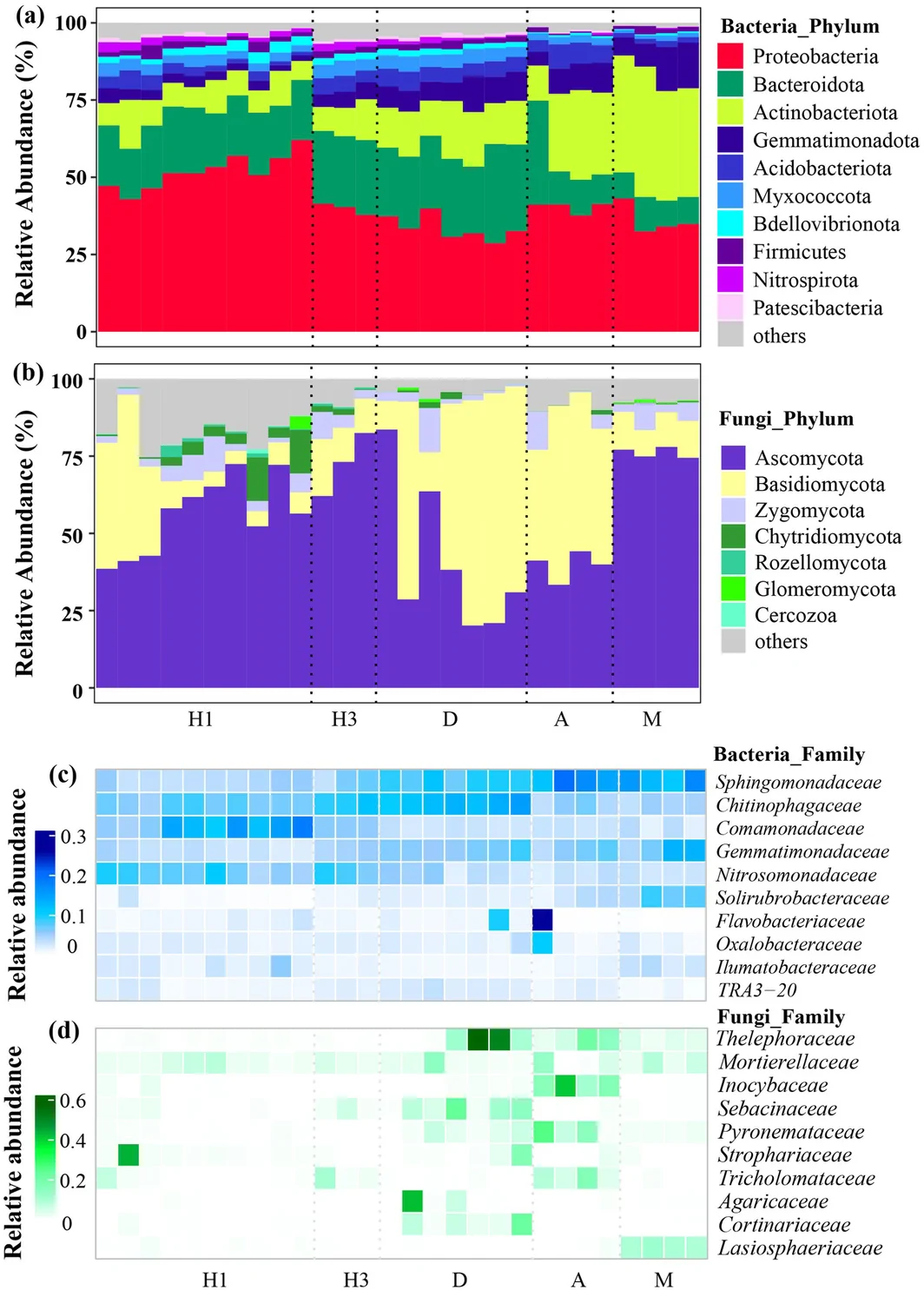
Dominant bacterial and fungal taxa in supraglacial environments from different glaciers. Relative abundance of dominant bacterial phyla (a). Relative abundance of dominant fungal phyla (b). Relative abundance of the top 10 dominant bacterial families (c). Relative abundance of the top 10 dominant fungal families (d).

Across the 28 supraglacial debris samples, a range of 41,903 ~ 72,807 high‐quality 16S rRNA gene sequences and 38,793 ~ 63,337 high‐quality ITS sequences were obtained per sample (Supporting Informations, Tables S3 and S4). These sequences were clustered into 16,525 bacterial OTUs and 5457 fungal OTUs, respectively. Based on the rarefaction curves, the obtained 16S rRNA gene and ITS sequences adequately captured the bacterial and fungal community diversity within the debris samples (Supporting Informations, Figure S1). Overall, a substantial proportion of unidentified taxa was observed in the supraglacial debris (Supporting Informations, Figure S2). Within the identified taxa, Proteobacteria, Bacteroidota, and Actinobacteriota were the dominant bacterial phyla across all sites, with mean relative abundances of 42.11%, 19.33%, and 16.60%, respectively (Figure 3a). *Sphingomonadaceae*, *Chitinophagaceae*, and *Comamonadaceae* were the dominant bacterial families (Figure 3c). Ascomycota and Basidiomycota occupied about 83% of the total fungal composition (Figure 3b), whereas the *Thelephoraceae*, *Mortierellaceae*, and *Inocybaceae* were the most abundant three families (Figure 3d). Many of the dominant bacterial phyla in the supraglacial debris varied between mountains and glaciers (Figure 3a, Supporting Informations, Tables S5 and S6). Specifically, the abundance of both the Bacteroidota and Actinobacteriota show significant differences between Mt. Gongga and Mt. Gangrigabu; in particular, it observed an obviously higher abundance of Actinobacteria in the supraglacial environments of the Midui (M) and Azha (A) Glaciers located on Mt. Gangrigabu region (*p* < 0.05). As for fungi, the relative abundance of Rozellomycota and Cercozoa showed significant differences between Mt. Gongga and Mt. Gangrigabu (Figure 3b, Supporting Informations, Tables S5 and S6), it was observed that the Dagongba (D) and Azha Glaciers had lower relative abundances of Ascomycota but higher abundances of Basidiomycota compared with the other glaciers (*p* < 0.05).

**FIGURE 4:**
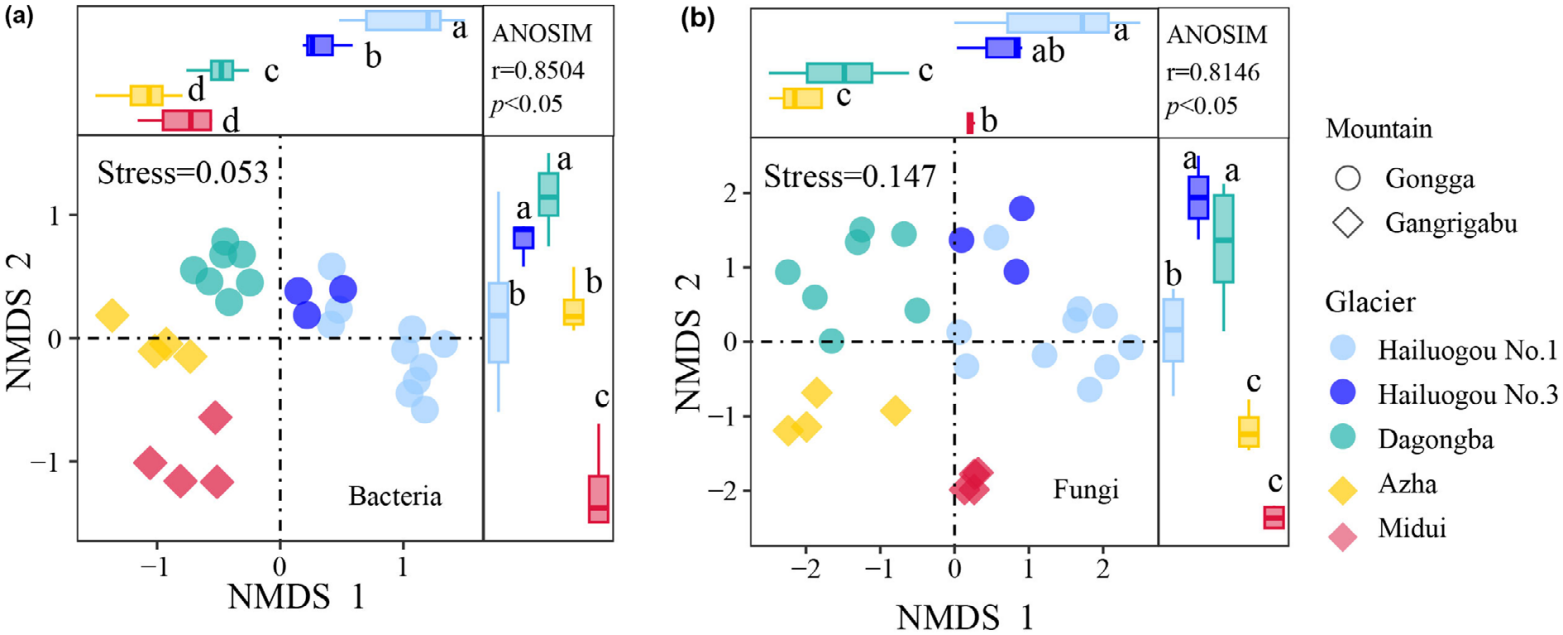
Nonmetric multidimensional scaling (NMDS) of bacterial (a) and fungal (b) community composition in the supraglacial environment from different glaciers. Each point represents a site, and sites are colored according to glacier, with different shapes indicating different mountains. The ANOSIM statistics (R and *p* values) are shown in the upper‐right corner of each panel. The small boxes along the upper and right margins show pairwise ANOSIM comparisons between glaciers. Different lowercase letters above the boxes indicate significant differences between groups (*p* < 0.05).

Non‐metric multidimensional scaling (NMDS) based on Bray–Curtis dissimilarity revealed clear clustering patterns of microbial communities among glaciers (Figure 4). ANOSIM further confirmed significant differences in community composition among glaciers for both bacteria (*R* = 0.8504, *p* < 0.05) and fungi (*R* = 0.8146, *p* < 0.05). Bacterial communities showed relatively tighter clustering within individual glaciers (Figure 4a), whereas fungal communities showed greater variation among sites (Figure 4b). Consistently, PERMDISP analysis indicated significant differences in within‐glacier dispersion for both bacterial (*F* = 5.05, *p* = 0.007) and fungal communities (*F* = 45.83, *p* = 0.001) (Supporting Informations, Table S7).

### 3.3 Alpha Diversity of Microbial Communities and Their Environmental Correlations

The alpha diversity indices of both bacterial and fungal communities varied significantly among glaciers from different mountain regions. For bacterial communities, both the Richness and Shannon indices were significantly higher in glaciers from the Gongga Mountain region compared to those from the Gangrigabu Mountain region (Table 1). Specifically, it observed a higher bacterial richness in supraglacial debris from Hailuogou No. 3 Glacier (H1), Dagongba Glacier (D), and Hailuogou No. 1 Glacier (H1) when compared with Azha Glacier (A) and Midui Glacier (M). A similar trend was observed in the bacterial Shannon index, with significantly higher bacterial diversity in the supraglacial environment from the Gongga Mountain region (9.67–10.46) than in the Gangrigabu Mountain region (7.45–8.80). For fungal communities, the alpha diversity showed less regional variation across different glaciers compared with bacterial communities.

**TABLE 1:** The alpha diversity of bacterial and fungal communities on supraglacial debris from different geographical locations.

	Glacier	Richness index	Shannon index
Bacteria	H1 (Gongga)	3513.05 (±660.21)b	9.67 (±0.52)b
	H3 (Gongga)	4572.7 (±217.67)a	10.46 (±0.1)a
	D (Gongga)	4373.44 (±296.82)a	10.25 (±0.27)a
	A (Gangrigabu)	2547.05 (±367.59)c	8.8 (±1.05)c
	M (Gangrigabu)	1153.2 (±289.35)c	7.45 (±0.68)c
Fungi	H1 (Gongga)	624.11 (±80.62)a	5.91 (±0.64)a
	H3 (Gongga)	550.07 (±73.23)ab	5.21 (±1.31)a
	D (Gongga)	453.03 (±105.58)ab	4.58 (±1.3)a
	A (Gangrigabu)	497.28 (±157.3)b	5.07 (±0.73)a
	M (Gangrigabu)	388.3 (±18.16)b	5.41 (±0.2)a

*Note:* Lowercase letters in different columns indicate that the corresponding indicators have significant differences among glaciers (K–W test, *p* < 0.05).

Spearman correlation analysis revealed that bacterial alpha diversity was strongly associated with supraglacial debris physicochemical properties (Table 2). Both bacterial richness and Shannon index were negatively correlated with pH (*r* = −0.682 and −0.653, *p* < 0.001) but positively correlated with TP (*r* = 0.472 and 0.368, *p* < 0.001) and NO_3_^−^–N (*r* = 0.825 and 0.804, *p* < 0.01) (Table 2). Fungal richness index showed a significant negative relationship with TP (*p* = −0.461, *p* < 0.01) and AP (*p* = −0.492, *p* < 0.01) (Table 2).

**TABLE 2:** Correlation between microbial alpha diversity and supraglacial debris properties.

	Bacteria		Fungi	
	Richness index	Shannon index	Richness index	Shannon index
Elevation	0.415	0.391	−0.353	−0.216
pH	−0.682***	−0.653***	−0.249*	0.115
TC	−0.363**	−0.295**	−0.462*	−0.369
TN	0.159	0.169	−0.328	−0.427
TP	0.472***	0.368***	0.312	0.289
TK	0.263	0.254	−0.461**	−0.352
OM	0.028	0.093	−0.325	−0.433
NO_3_^−^–N	0.825***	0.804**	0.03	−0.198
NH_4_^+^–N	−0.12	−0.159	0.167	−0.052
AN	0.114	0.183	−0.436	−0.388
AP	−0.445*	−0.393*	−0.271	−0.239
AK	−0.19	−0.19	−0.492**	−0.424

*Note:* Spearman's rank correlation coefficients (*p*) are presented in the table. Asterisks indicate the following significance thresholds: “*”, *p* < 0.05, “**”, *p* < 0.01; and “***”, *p* < 0.001.

### 3.4 The Environmental Determinants of Supraglacial Microbial Community Structures and Their Distance‐Decay Patterns

**FIGURE 5:**
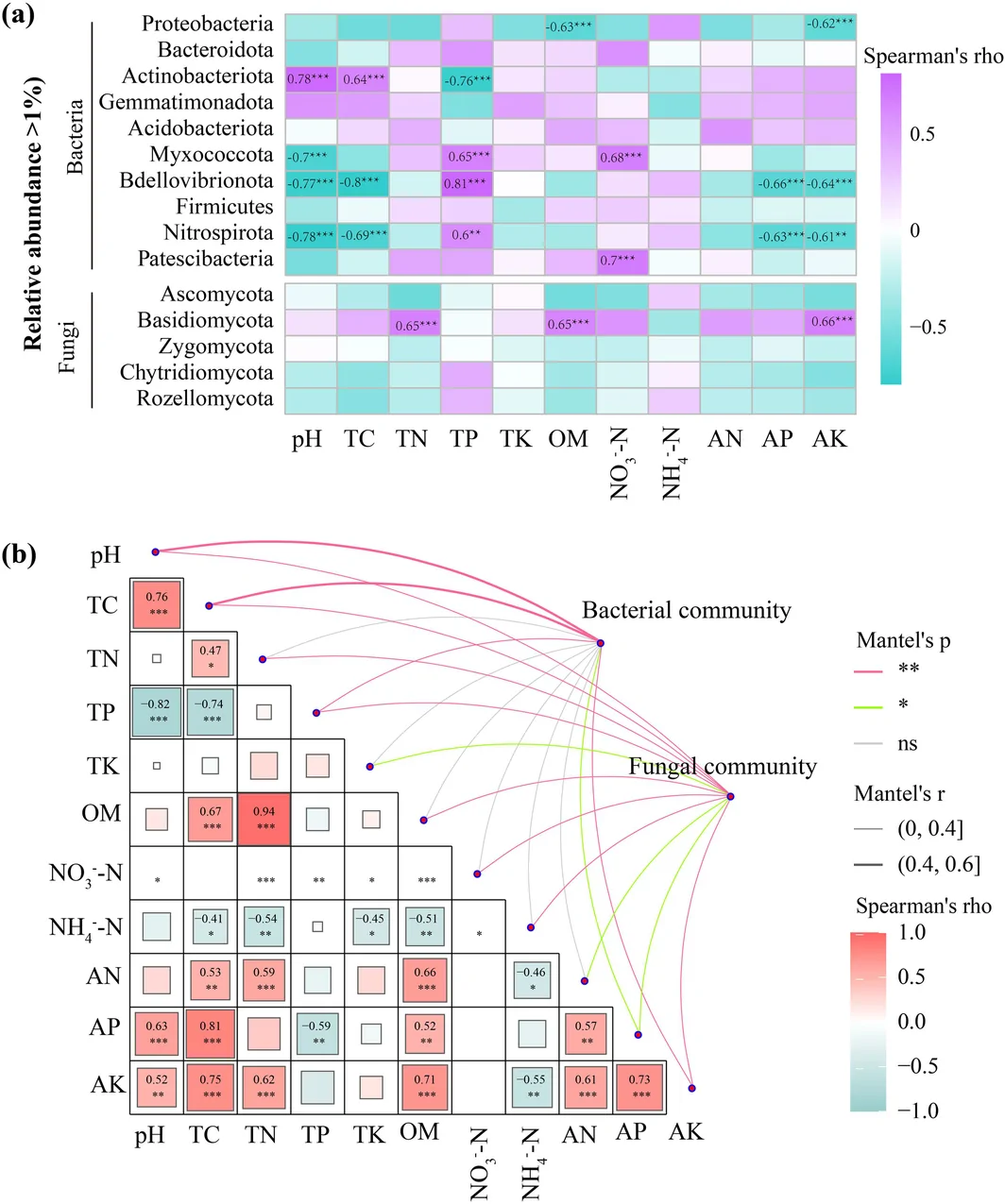
Influence of supraglacial debris properties on microbial abundance and community structure. a, Spearman correlation analysis between microbial abundance and physicochemical properties of supraglacial debris; b, Mantel test analysis of the relationships between bacterial and fungal community composition and supraglacial debris properties. In panel (a), colors indicate the direction of Spearman correlations (ρ), with different colors representing positive and negative correlations, and color intensity indicating the magnitude of the absolute correlation coefficient (|ρ|); darker colors indicate stronger correlations. The numbers in the heatmap cells represent Spearman's correlation coefficients (ρ). Asterisks indicate statistical significance (*p  < 0.05, **p  < 0.01, and ***p  < 0.001). In panel (b), the lower‐left heatmap shows Spearman correlations among physicochemical properties of supraglacial debris, with colors indicating the direction of correlations and color intensity representing the magnitude of |ρ|. The upper‐right links represent Mantel correlations between bacterial or fungal community composition and physicochemical properties. Colored links indicate significant correlations, with different colors representing different significance levels (*p  < 0.05 and **p  < 0.01), whereas non‐significant correlations are shown in gray. Link width represents the magnitude of the Mantel correlation coefficient (|ρ|).

The correlation analysis illustrates the potential links between the relative abundance of the top ten dominant bacterial and fungal phyla and the physicochemical properties of supraglacial debris (Figure 5a). Specifically, it found that the relative abundance of Proteobacteria was negatively correlated with OM (*R* = −0.63, *p* < 0.001) and TK content (*R* = −0.62, *p* < 0.001). Besides, Actinobacteria abundance positively associated with the pH value of supraglacial debris (*R* = 0.78, *p* < 0.001) and TC content (*R* = 0.64, *p* < 0.001). The abundance of Myxococcota (*R* = 0.65, *p* < 0.001), Bdellovibrionota (*R* = 0.81, *p* < 0.001), and Nitrospirota (*R* = 0.60, *p* < 0.01) were positively correlated with TP content of supraglacial debris. Additionally, Myxococcota (*R* = 0.68, *p* < 0.001) and Patescibacteria (*R* = 0.70, *p* < 0.001) were positively correlated with NO_3_^−^–N content of supraglacial debris. For fungi, only Basidiomycota showed significant positive correlations, specifically with TN (*R* = 0.65, *p* < 0.001), OM (*R* = 0.65, *p* < 0.001), and AK content (*R* = 0.66, *p* < 0.001). Mantel tests revealed that bacterial community composition was significantly shaped by supraglacial debris pH and TC content (Figure 5b).

**FIGURE 6:**
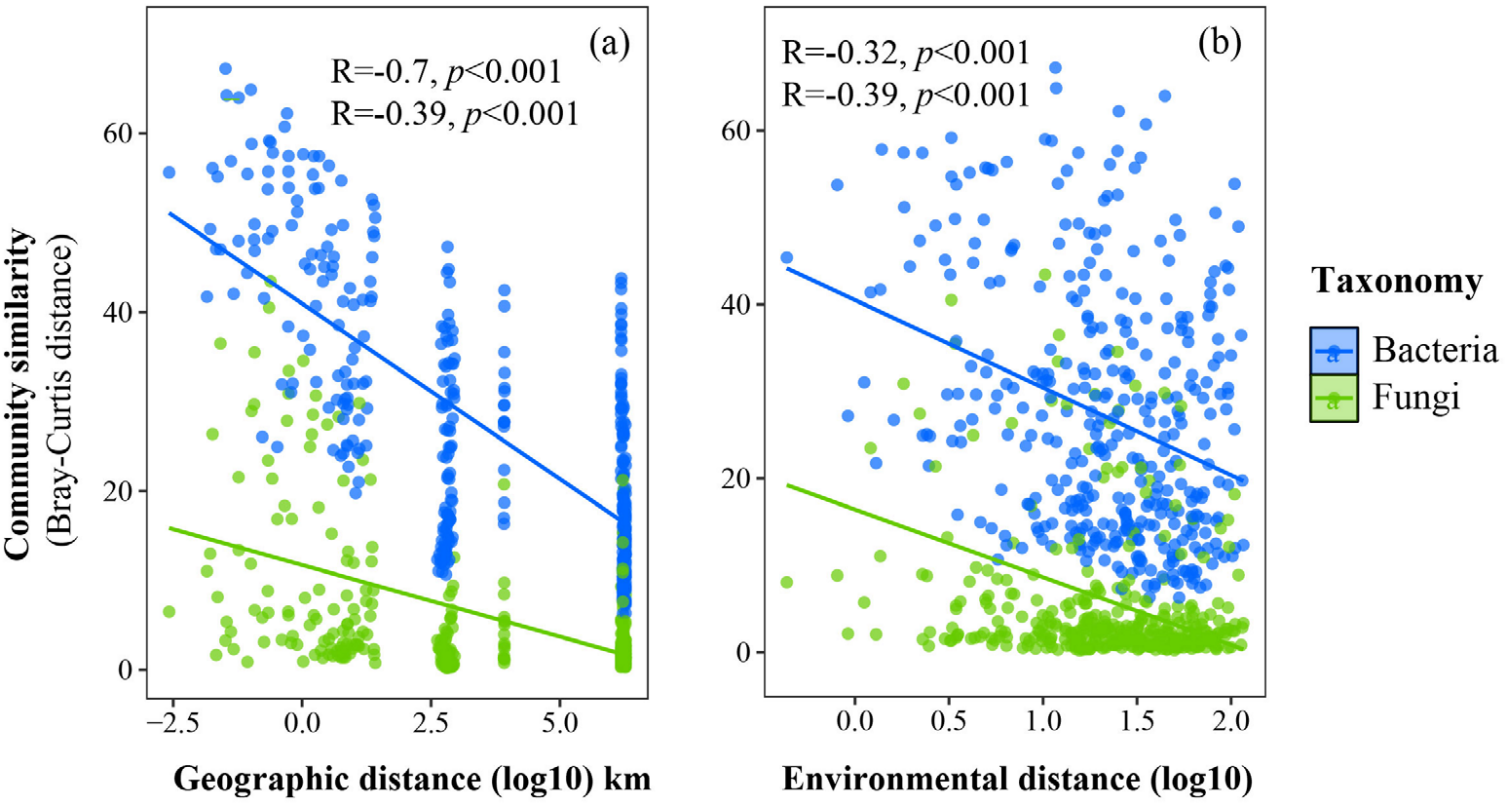
The relationship between the bacterial and fungal β‐diversity versus geographic distance (a) and environmental distance (b). The regression line denotes distance‐decay curves for the bacterial (blue point) and fungal (green point) communities.

To assess the spatial and environmental dependencies of microbial communities in supraglacial ecosystems, we examined the distance–decay relationships (DDRs) for bacterial and fungal assemblages across glacial debris samples (Figure 6). Bacteria displayed a significantly steeper DDR slope with geographic distance (slope = −0.70, *p* < 0.001) than fungi (slope = −0.39, *p* < 0.001), highlighting a greater sensitivity to spatial isolation. However, when related to environmental distance, the bacterial DDR slope (slope = −0.32, *p* < 0.001) weakened and became comparable to the fungal slope (slope = −0.39, *p* < 0.001).

**FIGURE 7:**
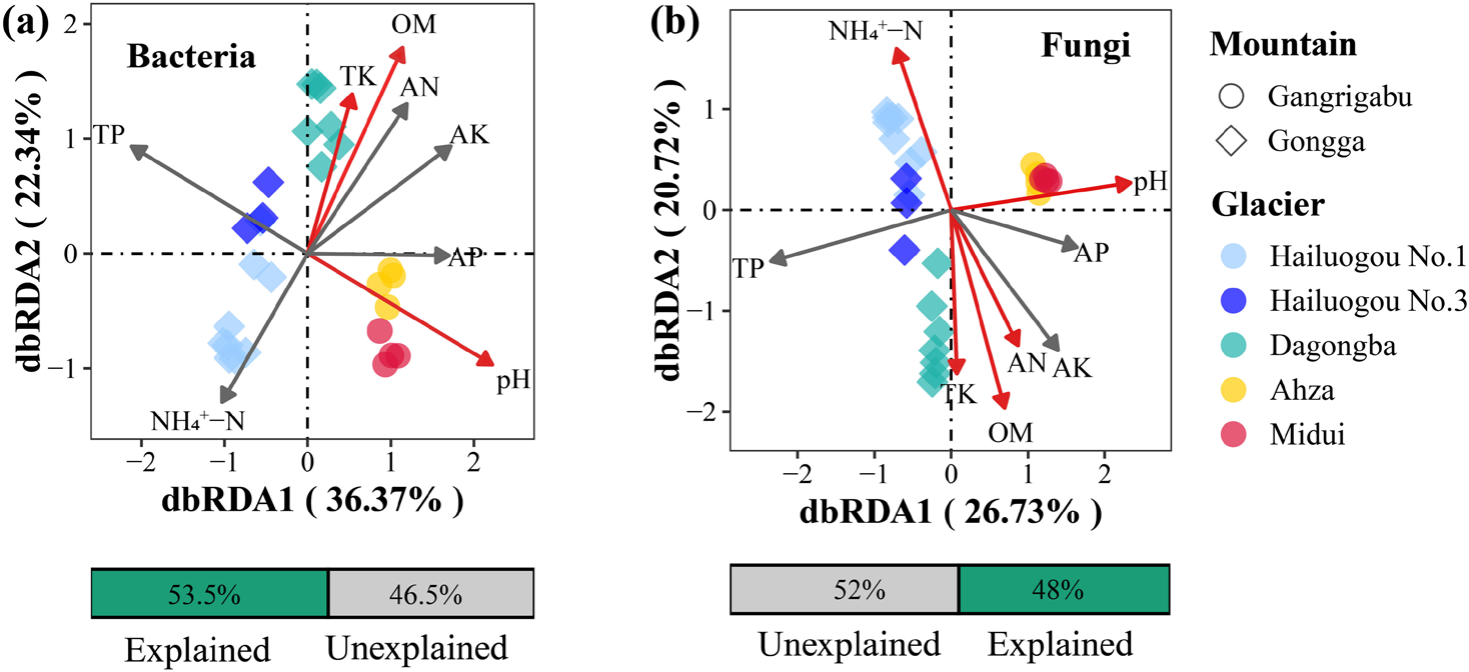
Distance‐based redundancy analysis (dbRDA) of bacterial (a) and fungal (b) community composition in relation to environmental variables. Samples are ordinated based on Bray–Curtis dissimilarities constrained by the measured environmental variables. Arrows represent environmental variables; their direction indicates the direction of increasing values, and their length reflects the strength of their correlations with community composition. Red arrows indicate environmental variables with significant effects identified by permutation tests (*p* < 0.05), whereas gray arrows represent non‐significant variables. The percentages on the axes indicate the proportion of constrained variation explained by each dbRDA axis. Each point represents a site, colors denote different glaciers (H1, H3, D, A, and M), and shapes distinguish the two glacier regions (Gongga and Gangrigabu). The stacked bar beneath each ordination summarizes the proportion of community variation explained by the measured environmental variables (green) and the unexplained variation (gray).

Distance‐based redundancy analysis (dbRDA) demonstrated that environmental variables significantly explained the variation in both bacterial (*F* = 2.59, *p* = 0.001) (Figure 7a) and fungal (*F* = 2.08, *p* = 0.001) (Figure 7b) community composition across the glaciers of Mountain Gongga and Gangrigabu, accounting for 53.5% (adjusted *R* ^2^ = 0.328) and 48.0% (adjusted *R* ^2^ = 0.249) of the total variation, respectively. For both bacterial and fungal communities, pH was the strongest environmental predictor, followed by organic matter (OM). In addition, total potassium (TK) was significantly associated with bacterial community variation, whereas total phosphorus (TP), total potassium (TK), ammonium nitrogen (NH_4_^+^‐N), and available nitrogen (AN) were identified as significant drivers of fungal community composition.

## 4 Discussion

### 4.1 Distance‐Decay Patterns of Microbial Communities Across Geographically Separated Glaciers

In this study, we investigated bacterial and fungal community diversity and biogeographic patterns across supraglacial debris layers on five debris‐covered glaciers from two different mountain regions, with the farthest geographic distance being around 530 km in the southeastern Tibetan Plateau. Consistent with the trends reported from other ecosystems, such as grasslands, forests, and agricultural soils (Liu et al. 2023, 2024; Zhu et al. 2024). This study observed a clear variation of microbial diversity across glaciers, both bacteria and fungi typically exhibit a clear distance‐decay relationship in community similarity (Figure 6), which illustrates that microbial composition becomes less similar as the geographical distance between sites is increased (Morlon et al. 2008; Liu et al. 2023). Consistently, on water‐filled microhabitats on glacier surfaces, which are called cryoconite holes, the microbiomes show pronounced distance‐decay patterns across glaciers from different locations (Ambrosini et al. 2017; Pittino et al. 2018; Pittino et al. 2023; Smith et al. 2018), with communities becoming increasingly dissimilar across spatial scales of just tens to hundreds of kilometers (Darcy et al. 2018; Liu et al. 2017). Besides, they demonstrated that cryoconite holes function as ecological “islands”, where liquid water and sediment inside the holes feed unique microbial communities, and the solid ice between them acts as an effective barrier to microbial dispersal (Darcy et al. 2018).

Although geographic isolation may constrain microbial dispersal to some extent, our community assembly analysis revealed that heterogeneous selection was the predominant process shaping both bacterial and fungal communities, whereas dispersal limitation played a relatively minor role (Supporting Information, Figure S3). These findings suggest that the distance‐decay pattern observed in glacier microbiomes is mainly associated with environmental heterogeneity among geographically separated glaciers, which promotes divergent microbial selection and community turnover. Previous studies found that even within seemingly homogeneous glacier surfaces, such as the Greenland Ice Sheet, there persists strong spatial heterogeneity that is driven by localized biological inputs and small‐scale physicochemical variability (Cameron et al. 2016; Graco‐Roza et al. 2022; Peguero et al. 2022). Previous studies on cryoconite holes demonstrated that pH and meltwater oxygen concentration of individual glaciers show significant differences and determined the microbial composition (Pittino et al. 2023). Besides, research on global glacier‐fed stream microbiomes exhibits strong biogeographic structuring, with bacterial communities differing markedly between mountain ranges and hemispheres due to geographic isolation and environmental selection (Ezzat et al. 2025).

We found that bacterial communities in supraglacial debris displayed a more pronounced distance‐decay pattern across glaciers than fungal communities (Figure 6), which indicated a fast turnover of bacterial composition in spatially separated mountain glaciers. This pattern is consistent with the study by Yao et al. (2025), which shows that in regional soil ecosystems, bacterial similarity declines more rapidly with geographic distance than that of fungi, as well as consistent with previous findings in soil microbial communities across region‐scale Taiga ecosystems (Liu et al. 2024). In the soil ecosystem, different microbial domains were shaped by different environmental factors and followed distinct community assembly patterns (Gong et al. 2023). In particular, bacterial composition has been widely reported to be strongly influenced by pH and nutrient content (Ni et al. 2021; Zhou et al. 2024). Consistently, our distance‐based redundancy analysis (dbRDA) demonstrated that environmental variables significantly shaped both bacterial and fungal community composition across the Mountain Gongga and Gangrigabu range, with pH and OM consistently emerging as the predominant driver for both bacterial and fungal groups (Figure 7). As we previously knew from Franzetti et al. (2013) and Hu et al. (2023), the rock material covering the glacier surface is largely derived from surrounding mountain slopes, which exhibit diverse rock types and parent material across different geographic regions. The nutrient profiles of supraglacial debris differed markedly among mountain regions (Figure 2). Besides, a thick and stable debris layer allowed the accumulation of more C and N (Hu et al. 2023). Usually, fungal communities often exhibit steeper distance‐decay slopes than bacteria in many other environments (Wang et al. 2017; Perini et al. 2019; Kang et al. 2023). They are primarily shaped by vegetation‐related factors, such as plant composition and litter inputs (Hu et al. 2023). This study found that fungal communities show weaker spatial turnover and a less pronounced distance‐decay relationship, because supraglacial debris from different glaciers generally barren landscape that lacks specific plant communities. The stronger distance‐decay of bacteria observed in supraglacial debris environment in this study may represent a habitat‐specific phenomenon, shaped by the unique habitat patchiness characteristic of supraglacial environments, rather than a general rule applicable to all ecosystems.

### 4.2 Determinants of Microbial Diversity on Debris‐Covered Glaciers

A previous large‐scale survey of bacterial communities across glaciers in China revealed that regional differences in temperature and precipitation were major drivers of bacterial community variation, highlighting the critical role of climate in shaping glacier microbial assemblages (Liu et al. 2015). The present study focuses on the Mt. Gangrigabu and Mt. Gongga region, both located on the southeast Tibetan Plateau, both influenced by monsoon climate (Fugger et al. 2022). At finer spatial scales where glaciers experience similar regional climatic regimes, local environmental heterogeneity may become a more important determinant of microbial community assembly. This study found that the bacterial diversity in supraglacial debris environments is sensitive to physicochemical conditions, particularly pH, C, and N gradients. In particular, the phylum of Actinobacteria was enriched in the supraglacial environments of Midui and Azha Glaciers (Figure 4), where relatively higher pH levels were recorded (Figure 2). The preferential enrichment of Actinobacteria in sites with higher pH observed in this study can be attributed to both their intrinsic physiological adaptations and the selective pressure imposed by alkaline conditions. Actinobacteria comprize a substantial number of alkaliphilic and alkali‐tolerant lineages that have evolved specialized mechanisms to thrive under high‐pH environments, including robust intracellular pH homeostasis mediated by Na^+^/H^+^ antiporter systems and cell wall buffering capacities (Baptista et al. 2022; Sorokin et al. 2022). Multiple laboratory and field studies from soil ecosystems also indicate that pH exerts a strong control on microbial community assembly through effects on mineral dissolution and nutrient availability (Ni et al. 2021; Zhou et al. 2024).

This study found that the *Comamonadaceae*, *Chitinophagaceae*, and *Micrococcaceae* dominate supraglacial habitats across both the Gongga and Gangrigabu mountain regions. The supraglacial environment has been widely reported to be dominated by rock weathering‐related taxa such as *Polaromonas* (Family: *Comamonadaceae*), *Sphingomonas*, *Angustibacter* (Franzetti et al. 2020; Kumar et al. 2022), and other cold‐adapted lineages. Given that supraglacial debris is primarily derived from local bedrock and surrounding mountain slopes, bedrock properties can influence supraglacial debris communities primarily through weathering‐derived chemical inputs that modify local physicochemical conditions (Lee et al. 2023). The weathering of minerals can release cations (e.g., Ca^2+^, Mg^2+^, K^+^) and affect meltwater pH and nutrient availability, thereby shaping microbial habitat suitability (Mitchell et al. 2013). Besides, biotic drivers, such as the abundance of Chironomidae Larvae, the predators of microbes, also influence the microbial diversity (Heather et al. 2024). For fungal taxa, this study found that the families such as *Thelephoraceae* (Phylum: Basidiomycota) and *Inocybaceae* (Phylum: Basidiomycota) were dominant on supraglacial debris. They are usually observed in nutrient‐poor terrains such as the barren land of glacier forefields and play a role in early‐stage rock weathering (Timling et al. 2012; Franzetti et al. 2020; de Vries et al. 2021; Szedlacsek et al. 2025). The dark, warm debris layer on the ice surface provides a refuge for fungal spores from the periglacial environment.

### 4.3 Conservation Implications of Microbial Diversity on Debris‐Covered Glaciers Under Climate Change

Our investigation of microbial communities across debris‐covered glaciers on the southeastern Tibetan Plateau reveals significant conservation implications. These supraglacial environments represent unique and functionally active habitats, with microbial communities exhibiting pronounced glacier specificity and strong distance‐decay patterns, indicating that each glacier may host distinct biotic assemblages. This makes individual glaciers unique reservoirs of microbial genomic diversity. As dynamic, climate‐sensitive refugia (Fickert et al. 2022), supraglacial debris habitats are acutely vulnerable to warming. Therefore, we argue that debris‐covered glaciers must be clearly documented and integrated into mountain biodiversity monitoring networks and regional conservation planning frameworks, which are essential not only for preserving unique microbial lineages but also for understanding the mechanisms of adaptation and evolutionary processes in alpine life.

## 5 Conclusion

In summary, this study found that debris‐covered glaciers harbor diverse bacterial and fungal communities, soil‐dwelling bacterial taxa such as *Sphingomonadaceae*, *Chitinophagaceae*, and *Comamonadaceae*, and fungal taxa such as *Thelephoraceae*, *Mortierellaceae*, and *Inocybaceae* were the dominants. Besides, there is a great variation of both microbial alpha and beta diversity in the supraglacial debris from different mountain regions, which was explained well by pH and available nitrogen content of supraglacial debris. Moreover, both bacterial and fungal communities showed strong distance‐decay patterns across geographically separated glaciers, which were mainly driven by heterogeneous selection associated with inter‐glacier environmental filtering, with pH serving as the dominant factor shaping microbial community differentiation. It seems that each glacier hosts specific microbial communities; local adaptation of microbes to the glacier micro‐environment plays important roles in shaping supraglacial microbial spatial patterns.

## Author Contributions

**Yang Hu:** data curation (equal), formal analysis (equal), investigation (equal), methodology (equal), validation (equal), visualization (equal), writing – original draft (equal). **Qiao Liu:** data curation (equal), funding acquisition (equal), investigation (equal). **Xuyang Lu:** conceptualization (equal), project administration (equal), resources (equal), supervision (equal), writing – review and editing (equal).

## Funding

This work was supported by the National Science Foundation of China (42361144874), the China Postdoctoral Science Foundation (2025M780331), and the Sichuan Science and Technology Program (2026NSFSC1162).

## Conflicts of Interest

The authors declare no conflicts of interest.

## Supporting information


**Table S1:** Primer sets used in PCR amplification.
**Table S2:** The reaction system and thermal profiles used in PCR amplification.
**Table S3:** Summary of Bacterial 16S rRNA gene amplicon sequencing data processing and OTU clustering results.
**Table S4:** Summary of Fungal ITS1 amplicon sequencing data processing and OTU clustering results.
**Table S5:** Statistical differences in microbial relative abundance at the phylum level between the two Mountains.
**Table S6:** Statistical differences in microbial relative abundance at the phylum level between the five glaciers.
**Table S7:** Homogeneity of multivariate dispersion (PERMDISP) of microbial communities among glaciers.
**Figure S1:** Rarefaction curves of bacterial (a) and fungal (b) communities based on the Shannon diversity index for supraglacial debris samples.
**Figure S2:** Taxonomic annotation rate of bacterial (a) and fungal (b) sequences at the genus level for supraglacial debris samples.
**Figure S3:** Relative contributions of different microbial community assembly processes based on the iCAMP framework.

## Data Availability

The raw sequence data reported in this paper are available in the NCBI (https://trace.ncbi.nlm.nih.gov/Traces/sra/) Sequence Read Archive under BioProject PRJNA1382505 and PRJNA1382518 for bacteria and fungi, respectively. The data that support the findings of this study are available on https://data.mendeley.com/datasets/xd8t34wxh9/1.
