# Supplementary material for: Cross‐Range Comparison of Supraglacial Microbial Communities Between Gongga and Gangrigabu Mountains, Southeast Tibetan Plateau
Source: Ecol Evol. 2026 Sep 6;16(9):e74278. doi: 10.1002/ece3.74278 (PMC13546813; doi:10.1002/ece3.74278)
Supplement: Supplementary file 1 [file ECE3-16-e74278-s001.docx]

**Table S1.** Primer sets used in PCR amplification

| Target group | Primer | Sequence (5′-3′) |
| --- | --- | --- |
| Bacterial 16S rRNA | 343F | TACGGRAGGCAGCAG |
|  | 798R | AGGGTATCTAATCCT |
| Fungal ITS | ITS1F | CTTGGTCATTTAGAGGAAGTAA |
|  | ITS2 | GCTGCGTTCTTCATCGATGC |

**Table S2.** The reaction system and thermal profiles used in PCR amplification

|  | Reaction system and volume | Temperature | Time | Cycle number |
| --- | --- | --- | --- | --- |
| First round PCR reaction | 2×Gflex PCR Buffer 15 μl  5 pmol/μl primer F 1 μl  5 pmol/μl primer R 1 μl  **Template DNA** ≥ 1 μl (50 ng)  Tks Gflex DNA Polymerase (1.25U/μl) 0.6 μl  H_2_O Add to 30 μl  **Total 30 μl** | 94 ℃ | 5 min | 1 |
|  |  | 94 ℃ | 30 S | 26 |
|  |  | 56 ℃ | 30 S |  |
|  |  | 72 ℃ | 20 S |  |
|  |  | 72 ℃ | 5 min | 1 |
|  |  | 4 ℃ | hold | - |
|  |  |  |  |  |
| Second round PCR reaction | 2×Gflex PCR Buffer 15 μl  Tks Gflex DNA Polymerase (1.25 U/μl 0.6 μl  Adapter I5 1 μl  Adapter I7 1 μl  **First PCR product** ≥ 1 μl (50 ng)  H_2_O Add to 30 μl  **Total 30 μl** | 94 ℃ | 5 min | 1 |
|  |  | 94 ℃ | 30 S | 7 |
|  |  | 56 ℃ | 30 S |  |
|  |  | 72 ℃ | 20 S |  |
|  |  | 72 ℃ | 5 min | 1 |
|  |  | 4 ℃ | hold | - |
|  |  |  |  |  |

**Table S3** Summary of Bacterial 16S rRNA gene amplicon sequencing data processing and OTU clustering results

| Sample_ID | clean_tags | valid_tags | Valid  percent | valid minLength | valid meanLength | valid maxLength | OTU  counts |
| --- | --- | --- | --- | --- | --- | --- | --- |
| HLG_01 | 71553 | 54971 | 76.83% | 229 | 416.89 | 452 | 5266 |
| HLG_02 | 72321 | 53982 | 74.64% | 236 | 415.82 | 442 | 4672 |
| HLG_03 | 73510 | 66101 | 89.92% | 246 | 416.23 | 452 | 4176 |
| HLG_04 | 70950 | 53777 | 75.80% | 254 | 419.39 | 442 | 3409 |
| HLG_05 | 65674 | 50092 | 76.27% | 258 | 418.56 | 442 | 3566 |
| HLG_06 | 70595 | 51172 | 72.49% | 236 | 418.8 | 452 | 3932 |
| HLG_07 | 70125 | 55487 | 79.13% | 259 | 419.27 | 452 | 3416 |
| HLG_08 | 71789 | 54843 | 76.39% | 256 | 418.09 | 446 | 3651 |
| HLG_09 | 71293 | 55212 | 77.44% | 236 | 417.93 | 452 | 3156 |
| HLG_10 | 70429 | 52434 | 74.45% | 250 | 418.96 | 442 | 2839 |
| HLG_11 | 71728 | 54181 | 75.54% | 5 | 417.53 | 452 | 4832 |
| HLG_12 | 74070 | 54886 | 74.10% | 259 | 416.66 | 450 | 5265 |
| HLG_13 | 69631 | 53061 | 76.20% | 236 | 415.65 | 451 | 4766 |
| DGB_01 | 72163 | 51895 | 71.91% | 227 | 415.94 | 458 | 4645 |
| DGB_02 | 73571 | 57018 | 77.50% | 256 | 415.4 | 451 | 5133 |
| DGB_03 | 72205 | 56821 | 78.69% | 228 | 414.69 | 450 | 5291 |
| DGB_04 | 70003 | 41903 | 59.86% | 229 | 414.18 | 451 | 3985 |
| DGB_05 | 70855 | 53328 | 75.26% | 228 | 415.53 | 442 | 4716 |
| DGB_06 | 70753 | 53743 | 75.96% | 228 | 416.05 | 451 | 4494 |
| DGB_07 | 71954 | 55870 | 77.65% | 228 | 415 | 452 | 4682 |
| AZ_01 | 76079 | 68615 | 90.19% | 229 | 417.34 | 452 | 2377 |
| AZ_02 | 75487 | 69813 | 92.48% | 229 | 412.54 | 452 | 2832 |
| AZ_03 | 76350 | 70514 | 92.36% | 282 | 413.09 | 451 | 3165 |
| AZ_04 | 76851 | 72078 | 93.79% | 229 | 413.51 | 442 | 3225 |
| MD_01 | 73972 | 71205 | 96.26% | 259 | 413.41 | 459 | 1193 |
| MD_02 | 74255 | 70626 | 95.11% | 257 | 412.81 | 451 | 1113 |
| MD_03 | 74641 | 71257 | 95.47% | 229 | 414.77 | 452 | 1767 |
| MD_04 | 76060 | 72807 | 95.72% | 245 | 413.33 | 442 | 1173 |

**Table S4** Summary of Fungal ITS1 gene amplicon sequencing data processing and OTU clustering results

| Sample_ID | clean_tags | valid_tags | Valid  percent | valid minLength | valid meanLength | valid maxLength | OTU  counts |
| --- | --- | --- | --- | --- | --- | --- | --- |
| HLG_01 | 52616 | 52206 | 99.22% | 158 | 268.71 | 366 | 644 |
| HLG_02 | 51399 | 50829 | 98.89% | 163 | 280.97 | 366 | 602 |
| HLG_03 | 58732 | 57901 | 98.59% | 158 | 258.48 | 363 | 863 |
| HLG_04 | 39513 | 38793 | 98.18% | 166 | 262.95 | 364 | 624 |
| HLG_05 | 55120 | 54615 | 99.08% | 166 | 246.46 | 368 | 659 |
| HLG_06 | 51300 | 50875 | 99.17% | 158 | 247.7 | 366 | 645 |
| HLG_07 | 54758 | 54306 | 99.17% | 163 | 264.49 | 361 | 614 |
| HLG_08 | 54604 | 54169 | 99.20% | 167 | 246.58 | 365 | 553 |
| HLG_09 | 53209 | 52820 | 99.27% | 167 | 274.58 | 366 | 566 |
| HLG_10 | 51117 | 50652 | 99.09% | 161 | 269.97 | 361 | 715 |
| HLG_11 | 51419 | 50983 | 99.15% | 165 | 249.7 | 363 | 577 |
| HLG_12 | 56825 | 56256 | 99.00% | 163 | 256.61 | 358 | 670 |
| HLG_13 | 41382 | 41071 | 99.25% | 160 | 242.07 | 354 | 479 |
| DGB_01 | 56734 | 56365 | 99.35% | 158 | 269.91 | 355 | 406 |
| DGB_02 | 57562 | 57102 | 99.20% | 158 | 277.19 | 364 | 577 |
| DGB_03 | 56716 | 56179 | 99.05% | 158 | 252.47 | 350 | 664 |
| DGB_04 | 59803 | 59351 | 99.24% | 164 | 266.26 | 366 | 542 |
| DGB_05 | 58070 | 57595 | 99.18% | 163 | 271.19 | 363 | 418 |
| DGB_06 | 51293 | 50831 | 99.10% | 167 | 272.08 | 358 | 376 |
| DGB_07 | 58578 | 58024 | 99.05% | 167 | 284.86 | 362 | 455 |
| AZ_01 | 40162 | 39606 | 98.62% | 158 | 272.38 | 365 | 395 |
| AZ_02 | 56468 | 55876 | 98.95% | 158 | 283.06 | 364 | 412 |
| AZ_03 | 45357 | 44653 | 98.45% | 173 | 275.59 | 364 | 528 |
| AZ_04 | 45953 | 45189 | 98.34% | 158 | 275.27 | 364 | 733 |
| MD_01 | 63576 | 63040 | 99.16% | 158 | 243.98 | 358 | 426 |
| MD_02 | 63882 | 63337 | 99.15% | 158 | 241.25 | 356 | 388 |
| MD_03 | 58634 | 58095 | 99.08% | 158 | 243.58 | 368 | 384 |
| MD_04 | 60406 | 59739 | 98.90% | 158 | 242.77 | 361 | 404 |

**Table S5** Statistical differences in microbial relative abundance

at the phylum level between the two Mountains

|  | **Taxonomy** | **Mountain** | | **p value** |
| --- | --- | --- | --- | --- |
|  |  | **Gangrigabu** | **Gongga** |  |
| **Bacteria** | Proteobacteria | 0.383 ± 0.014 | 0.437 ± 0.022 | 0.284 ns |
|  | Bacteroidota | 0.128 ± 0.030 | 0.220 ± 0.008 | 0.002** |
|  | **Actinobacteriota** | 0.303 ± 0.034 | 0.111 ± 0.009 | <0.001*** |
|  | Gemmatimonadota | 0.092 ± 0.013 | 0.050 ± 0.006 | 0.006** |
|  | Acidobacteriota | 0.039 ± 0.009 | 0.040 ± 0.004 | 0.901 ns |
|  | Myxococcota | 0.018 ± 0.002 | 0.037 ± 0.002 | <0.001*** |
|  | Bdellovibrionota | 0.006 ± 0.001 | 0.022 ± 0.002 | <0.001*** |
|  | Firmicutes | 0.012 ± 0.003 | 0.019 ± 0.002 | 0.026* |
|  | Nitrospirota | 0.002 ± 0.001 | 0.016 ± 0.002 | <0.001*** |
|  | Patescibacteria | 0.003 ± 0.001 | 0.008 ± 0.001 | 0.001** |
| **Fungi** | Ascomycota | 0.579 ± 0.070 | 0.532 ± 0.044 | 0.654 ns |
|  | Basidiomycota | 0.291 ± 0.072 | 0.280 ± 0.059 | 0.711 ns |
|  | Zygomycota | 0.047 ± 0.015 | 0.043 ± 0.008 | 0.940 ns |
|  | Chytridiomycota | 0.003 ± 0.002 | 0.028 ± 0.009 | 0.114 ns |
|  | Rozellomycota | 0.000 ± 0.000 | 0.005 ± 0.002 | 0.046* |
|  | Glomeromycota | 0.002 ± 0.001 | 0.003 ± 0.002 | 0.498 ns |
|  | Cercozoa | 0.000 ± 0.000 | 0.001 ± 0.001 | 0.023* |

Values represent mean ± standard deviation (SD) of relative abundance. P-values were calculated using the two-tailed Wilcoxon rank-sum test to compare differences between the two mountains. Significance levels are indicated as follows: “*”, P < 0.05, “**”, P < 0.01, “***”, P < 0.001. NS indicates not significant.

**Table S6** Statistical differences in microbial relative abundance at the phylum level between the five glaciers

|  | **Taxonomy** | **Glacier** | | | | |
| --- | --- | --- | --- | --- | --- | --- |
|  |  | **H1(Gongga)** | **H3(Gongga)** | **D(Gongga)** | **A(Gangrigabu)** | **M(Gangrigabu)** |
| **Bacteria** | Proteobacteria | 0.519 ± 0.018b | 0.399 ± 0.010ab | 0.335 ± 0.015a | 0.404 ± 0.009ab | 0.362 ± 0.024a |
|  | Bacteroidota | 0.192 ± 0.006bc | 0.236 ± 0.003ab | 0.251 ± 0.014a | 0.163 ± 0.058abc | 0.092 ± 0.007c |
|  | **Actinobacteriota** | **0.089 ± 0.009c** | 0.101 ± 0.016ac | 0.147 ± 0.010ab | **0.230 ± 0.040ab** | **0.377 ± 0.016b** |
|  | Gemmatimonadota | 0.030 ± 0.003b | 0.049 ± 0.004ab | 0.079 ± 0.008a | 0.077 ± 0.011a | 0.106 ± 0.023a |
|  | Acidobacteriota | 0.030 ± 0.005bc | 0.048 ± 0.005abc | 0.051 ± 0.003ab | 0.060 ± 0.009a | 0.017 ± 0.001c |
|  | Myxococcota | 0.032 ± 0.002ac | 0.044 ± 0.004c | 0.043 ± 0.002c | 0.023 ± 0.000ab | 0.013 ± 0.002b |
|  | Bdellovibrionota | 0.026 ± 0.003b | 0.022 ± 0.000b | 0.018 ± 0.001ab | 0.006 ± 0.001a | 0.005 ± 0.001a |
|  | Firmicutes | 0.021 ± 0.003a | 0.018 ± 0.003a | 0.018 ± 0.002a | 0.007 ± 0.003a | 0.016 ± 0.004a |
|  | Nitrospirota | 0.019 ± 0.002b | 0.022 ± 0.003b | 0.010 ± 0.002ab | 0.002 ± 0.001a | 0.002 ± 0.001a |
|  | Patescibacteria | 0.007 ± 0.001ab | 0.009 ± 0.000ab | 0.010 ± 0.001a | 0.004 ± 0.001ab | 0.001 ± 0.000b |
| **Fungi** | Ascomycota | 0.561 ± 0.039ab | 0.726 ± 0.059ab | 0.409 ± 0.090a | 0.397 ± 0.023a | 0.761 ± 0.008b |
|  | Basidiomycota | 0.166 ± 0.057b | 0.135 ± 0.024ab | 0.505 ± 0.105a | 0.473 ± 0.048ab | 0.110 ± 0.009ab |
|  | Zygomycota | 0.045 ± 0.010a | 0.053 ± 0.018a | 0.036 ± 0.018a | 0.044 ± 0.029a | 0.050 ± 0.015a |
|  | Chytridiomycota | 0.046 ± 0.016b | 0.013 ± 0.005ab | 0.007 ± 0.004ab | 0.004 ± 0.003a | 0.003 ± 0.000ab |
|  | Rozellomycota | 0.008 ± 0.003b | 0.007 ± 0.001b | 0.000 ± 0.000a | 0.000 ± 0.000a | 0.000 ± 0.000a |
|  | Glomeromycota | 0.004 ± 0.004a | 0.000 ± 0.000a | 0.003 ± 0.002a | 0.000 ± 0.000a | 0.005 ± 0.001a |
|  | Cercozoa | 0.002 ± 0.001a | 0.000 ± 0.000ab | 0.000 ± 0.000ab | 0.000 ± 0.000ab | 0.000 ± 0.000b |

Data are presented as mean ± standard deviation (SD) of relative abundance. Pairwise comparisons among glaciers were performed using Dunn's multiple comparison test with Benjamini-Hochberg (BH) false discovery rate (FDR) correction. Within each row, different lowercase letters indicate significant differences among glaciers (P < 0.05).

| **Glacier** | **Distance to centroid (mean ± SD)** | |
| --- | --- | --- |
|  | **Bacteria** | **Fungi** |
| H1 (n=10) | 0.390 ± 0.096 | 0.566 ± 0.026 |
| H3 (n=3) | 0.245 ± 0.035 | 0.458 ± 0.030 |
| D (n=7) | 0.313 ± 0.032 | 0.592 ± 0.015 |
| A (n=4) | 0.292 ± 0.078 | 0.416 ± 0.007 |
| M (n=4) | 0.287 ± 0.043 | 0.150 ± 0.005 |
| **PERMDISP test** | **F = 5.049, P = 0.007** | **F = 45.83, P = 0.001** |

**Table S7** Homogeneity of multivariate dispersion (PERMDISP) of microbial communities among glaciers

Values represent the mean ± SD of distances to centroid for bacterial and fungal communities. Bray-Curtis dissimilarity matrices were used to calculate multivariate dispersion, and differences among glaciers were tested using PERMDISP (betadisper) with permutation tests (999 permutations). F-values and P-values were obtained from the permutation-based test of homogeneity of multivariate dispersions.

**Figure S1** Rarefaction curves of bacterial (a) and fungal (b) communities based on the Shannon diversity index for supraglacial debris samples.

**Figure S2** Taxonomic annotation rate of bacterial (a) and fungal (b) sequences at the genus level for supraglacial debris samples.

**Figure S3** Relative contributions of different microbial community assembly processes based on the iCAMP framework. Pie charts show the relative contributions of five ecological processes for bacterial (a) and fungal (b) communities,including heterogeneous selection (HeS), homogeneous selection (HoS), dispersal limitation (DL), homogenizing dispersal (HD), and undominated processes (UP). Ecological processes were inferred using the qpen function in the iCAMP R package, based on community abundance and phylogenetic distance matrices, with 500 (bacteria) and 1,000 (fungi) randomizations. Process assignment followed thresholds of |βNTI| = 2 and |RCbray| = 0.95: βNTI > 2, HeS; βNTI < −2, HoS; |βNTI| ≤ 2 and RCbray > 0.95, DL; RCbray < −0.95, HD; |RCbray| ≤ 0.95, UP. Percentages indicate proportions of pairwise sample comparisons assigned to each process.
